# Spectral Fingerprints of Cortical Neuromodulation

**DOI:** 10.1523/JNEUROSCI.1801-21.2022

**Published:** 2022-05-04

**Authors:** Angela Radetz, Markus Siegel

**Affiliations:** ^1^Department of Neural Dynamics and Magnetoencephalography, Hertie Institute for Clinical Brain Research, University of Tübingen, 72076 Tübingen, Germany; ^2^Werner Reichardt Centre for Integrative Neuroscience, University of Tübingen, 72076 Tübingen, Germany; ^3^MEG Center, University of Tübingen, 72076 Tübingen, Germany; ^4^Neuroimaging Center, University Medical Center of the Johannes Gutenberg-University Mainz, 55131 Mainz, Germany

**Keywords:** acetylcholine, human MEG, neural dynamics, neuromodulation, noradrenaline, pupil dynamics

## Abstract

Pupil size has been established as a versatile marker of noradrenergic and cholinergic neuromodulation, which has profound effects on neuronal processing, cognition, and behavior. However, little is known about the cortical control and effects of pupil-linked neuromodulation. Here, we show that pupil dynamics are tightly coupled to temporally, spectrally, and spatially specific modulations of local and large-scale cortical population activity in the human brain. We quantified the dynamics of band-limited cortical population activity in resting human subjects using magnetoencephalography and investigated how neural dynamics were linked to simultaneously recorded pupil dynamics. Our results show that pupil-linked neuromodulation does not merely affect cortical population activity in a stereotypical fashion. Instead, we identified three frontal, precentral, and occipitoparietal networks, in which local population activity with distinct spectral profiles in the theta, beta, and alpha bands temporally preceded and followed changes in pupil size. Furthermore, we found that amplitude coupling at ∼16 Hz in a large-scale frontoparietal network predicted pupil dynamics. Our results unravel network-specific spectral fingerprints of cortical neuromodulation in the human brain that likely reflect both the causes and effects of neuromodulation.

**SIGNIFICANCE STATEMENT** Brain function is constantly affected by modulatory neurotransmitters. Pupil size has been established as a versatile marker of noradrenergic and cholinergic neuromodulation. However, because the cortical correlates of pupil dynamics are largely unknown, fundamental questions remain unresolved. Which cortical networks control pupil-linked neuromodulation? Does neuromodulation affect cortical activity in a stereotypical or region-specific fashion? To address this, we quantified the dynamics of cortical population activity in human subjects using magnetoencephalography. We found that pupil dynamics are coupled to highly specific modulations of local and large-scale cortical activity in the human brain. We identified four cortical networks with distinct spectral profiles that temporally predicted and followed pupil size dynamics. These effects likely reflect both the cortical control and effect of neuromodulation.

## Introduction

**Figure 1.**
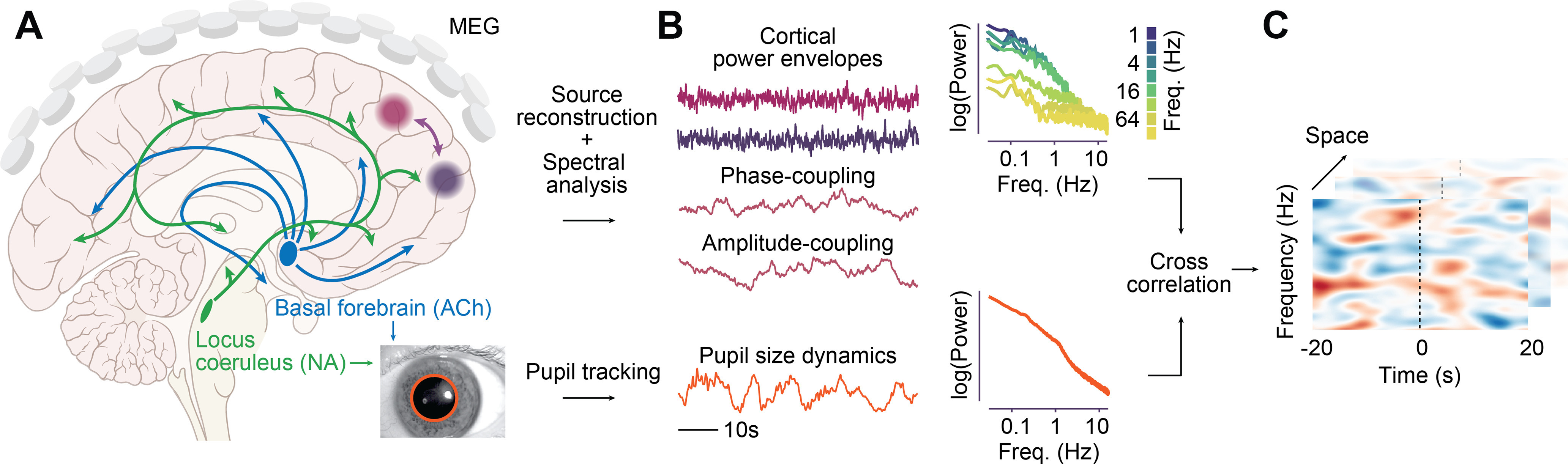
Research concept and approach. ***A***, LC-NA and BF-ACh systems with widespread neuromodulatory effects on neuronal population activity as measured with MEG. Pupil size reflects activity of both systems. ***B***, MEG data were source reconstructed and subjected to spectral analysis. Neuronal power envelopes and the dynamic of cortical phase and amplitude coupling were each temporally cross-correlated with pupil dynamics. Insets show power spectra of pupil size time sources and of power envelopes in different frequency bands averaged across all subjects and cortical space. ***C***, 3D cluster permutation statistics were used to identify coupling between neuronal and pupil dynamics across cortical space, frequency, and relative time lag between pupil and the neuronal dynamics.

Brain function, cognition and behavior are dynamically modulated by modulatory neurotransmitters such as noradrenaline (NA) and acetylcholine (ACh; Aston-Jones and Cohen, 2005; Yu and Dayan, 2005; Briand et al., 2007; Harris and Thiele, 2011; McGinley et al., 2015b). While ACh is predominantly released by the basal forebrain (BF), the major source of cortical NA is the locus coeruleus (LC), located in the dorsal pons (Fig. 1*A*). Both neurotransmitters have widespread, but locally specific effects on global brain networks and have been implicated in mediating arousal as driven by various factors such as surprise, attention, or uncertainty in cognition and behavior (Aston-Jones and Cohen, 2005; McGinley et al., 2015b).

In human research, no methods are available to directly track the impact of modulatory neurotransmitter release on neural processing. However, accumulating evidence suggests that changes in pupil size provide an indirect marker for noradrenergic and cholinergic neuromodulation (McDougal and Gamlin, 2015; Joshi and Gold, 2020). Activation of noradrenergic and cholinergic axons in sensory cortex predicts pupil size changes in mice (Reimer et al., 2016) and electrical stimulation of the LC robustly drives pupil dilations in nonhuman primates (Joshi et al., 2016). Complementary evidence is provided by human fMRI studies (Alnæs et al., 2014; Murphy et al., 2014; de Gee et al., 2017). Accordingly, a growing body of literature has linked modulations of pupil size to neuromodulation and changes in arousal, uncertainty, exploration–exploitation preference, engagement, and other factors in humans (Kahneman and Beatty, 1966; Einhäuser et al., 2008, 2010; Gilzenrat et al., 2010; Jepma and Nieuwenhuis, 2011; Preuschoff et al., 2011; Nassar et al., 2012; Alnæs et al., 2014; de Gee et al., 2014; Pajkossy et al., 2017; Urai et al., 2017).

However, despite the strong interest in pupil size as a measure of neuromodulation and arousal, little is known about the cortical correlates of neuromodulation. In animal studies, a tight link among pupil size, exploratory behaviors, and low-frequency fluctuations in membrane and local field potentials (LFPs) has been demonstrated, but only in a few selected brain regions (Reimer et al., 2014; Vinck et al., 2015; McGinley et al., 2015a,b). In humans, fMRI studies link pupil size to widespread changes in cortical activity (Yellin et al., 2015; van den Brink et al., 2016), but lack temporal resolution to identify the temporal relationship between pupil modulations and neuronal effects as well as to resolve the rich temporal structure of neuronal population activity. Recent electrophysiological studies in humans have identified pupil size-related population activity in selected brain regions and frequency ranges and have linked this activity to task-specific neuronal activity (Kucyi and Parvizi, 2020) and perceptual performance (Waschke et al., 2019; Podvalny et al., 2021). Despite these investigations, the relationship between cortical activity and pupil dynamics has not been directly quantified across the temporal, cortical, and spectral space. Thus, the cortical correlates of pupil-indexed neuromodulation remain largely unclear, and fundamental questions remain unresolved. Which cortical networks precede, and thus potentially control, pupil-linked neuromodulation? And does such neuromodulation affect cortical activity in a stereotypical or region-specific fashion?

Noradrenergic and cholinergic neuromodulation modulates neural gain (i.e., the responsiveness of units to received input) and may thus not only affect local population activity, but also large-scale cortical network interactions (Foote and Morrison, 1987; Servan-Schreiber et al., 1990; McCormick, 1992; Berridge and Waterhouse, 2003; Aston-Jones and Cohen, 2005; Kuo and Trussell, 2011; Pinto et al., 2013; Joshi et al., 2016). Furthermore, interactions within large-scale cortical networks may also be causally involved in controlling neuromodulation. However, similar to local population activity, little is known about the large-scale coupling of cortical activity in relation to pupil dynamics (Yellin et al., 2015).

To address this, we combined human magnetoencephalography (MEG) with simultaneous pupil-size recordings and systematically characterized the brainwide relationship between pupil dynamics and frequency-specific local cortical population activity as well as large-scale cortical coupling.

## Materials and Methods

### Participants and experimental procedure.

We acquired MEG data from 41 participants, who were seated in a dimly lit magnetically shielded chamber (Radetz and Siegel, 2018). A first sample of participants (*n* = 26) was instructed to fixate a dot in the middle of a screen at ∼60 cm viewing distance and to let their mind wander. The second sample (*n* = 15) was recorded under identical conditions, but without a fixation dot. Instead, participants were instructed to fixate the middle of the screen. The mean age of all subjects was 27 years with an SD of 4.1 years, and 25 subjects were female. The two samples did not differ regarding age or gender distribution (*p* > 0.05). The study was approved by the local ethics committee and conducted in accordance with the Declaration of Helsinki. Written informed consent was obtained from all participants before data acquisition.

### Electrophysiological recordings.

We recorded 10 min of MEG data per subject with a 275-channel whole-head system (Omega 2000, CTF Systems) at the MEG Center in Tübingen, Germany. The sampling rate of the recordings was 2343.75 Hz (antialiasing filter at one-half the Nyquist frequency). Head position was continuously measured using three head localization coils placed above the nasion and at the left and right preauricular points. Binocular gaze position and pupil size were recorded with an eyetracking system (Eyelink 1000, SR Research) with a sampling rate of 1000 Hz. Pupil size (area) and gaze position were fed into the MEG system and saved along with the MEG data. We recorded the electrooculogram (EOG) with electrodes placed vertically above and below the right eye, and horizontally at the outer canthus of each eye. Electrocardiography (EKG) was conducted by placing an electrode below the right clavicle as well as below the left lower costal arch. The ground electrode was placed on the left forearm.

### Data preprocessing.

All data were preprocessed using custom-built MATLAB scripts (MathWorks) and the FieldTrip toolbox (Oostenveld et al., 2011). All data were downsampled to 1000 Hz for further analysis. For MEG preprocessing, we used the preprocessing pipeline of the Human Connectome Project (Van Essen et al., 2013). In short, malfunctioning channels and data segments were identified and removed based on their correlation of the raw signal and signal variance with neighboring channels, *z*-scoring and thresholding of the data, and on iterative independent component analysis (ICA). We removed artifactual ICA components based on their sensor topography, spectrotemporal characteristic, and relation to the EOG and EKG using a semiautomatic procedure.

Gaze position and pupil size of each eye were thresholded to identify eye blinks, and a window of 1 s around eye blinks was marked. Next, all gaze positions and pupil size time courses were smoothed using a Savitzky–Golay filter with a third-order polynomial and a frame size of 0.7 s (∼1.5 Hz effective low-pass cutoff frequency). We calculated two time-dependent measures of eye movements: gaze position variance and gaze eccentricity. Gaze position variance was independently estimated using a 0.4 s sliding window for horizontal and vertical gaze positions and subsequently was averaged over both spatial dimensions. Gaze eccentricity was calculated from the gaze position for each time point and eye. We averaged pupil size, gaze position variance, and gaze eccentricity of the left and right eye except for three participants, for which only data from one eye were available. For all three resulting eye data time courses, we interpolated the time intervals marked as eyeblinks using a shape-preserving piecewise cubic interpolation. Finally, all eye data were downsampled to 143 Hz and *z* scored. Thus, all pupil time courses were rescaled to unit variance per recording session including slow drifts as well as fast pupil dynamics. We computed the power spectrum of pupil time courses across half-overlapping 100 s windows with Hanning tapering.

### MRI data acquisition.

For the first sample of subjects (*n* = 26), we acquired structural MRIs using a T1-weighted sagittal MP-RAGE sequence (TE = 2.18 ms, TR = 2300 ms, TI = 1100 ms, flip angle = 9°, 192 slices, voxel size = 1 × 1 × 1 mm³) and a Siemens MAGNETOM Trio 3 T scanner equipped with a 32-channel head coil. For the second sample (*n* = 15), T1-weighted sagittal MP-RAGE sequence data were available from previous measurements and thus acquired with slightly different acquisition protocols from different scanners. For all scans, the voxel size was 1 × 1 × 1 mm³ and the flip angle was 9°, and they consisted of 192 slices.

### Spectral analysis.

We computed spectral estimates for 35 frequencies ranging from 0.6 Hz (2^−0.75^) to 215 Hz (2^7.75^) in quarter-octave steps using Morlet's wavelets with a spectral bandwidth of 0.5 octaves (*f/*σ*_t_* = 5.8; kernels width, 5 σ*_t_*). Time–frequency estimates were computed with 143 Hz temporal resolution.

### Physical forward model and source projection.

For each subject, we constructed a single-shell head model (Nolte, 2003) based on the individual segmented MRI. MEG sensor positions and head model were aligned in the participant's head space, taking into account the continuously tracked head movements. We computed lead fields for 457 equally spaced (∼1.2 cm distance) source points spanning the cortex at 0.7 cm depth below the pial surface (Hipp et al., 2011). This source shell was generated in MNI space and nonlinearly transformed to individual headspace. We applied frequency–domain beamforming to reconstruct source-level activity (Van Veen et al., 1997; Gross et al., 2001). For each frequency and source location, we computed one filter, pointing in the dominant dipole orientation. We multiplied the frequency domain sensor-level MEG data with this filter for projection to the source level.

### Power envelopes and coupling measures.

For each frequency and cortical source, we computed power envelopes as the time course of log power. As for the pupil time courses, we computed the power spectrum of power envelopes in half-overlapping 100 s windows with Hanning tapering. We used pairwise measures of amplitude and phase coupling that are insensitive to field spread effects. For amplitude coupling, we computed amplitude envelope correlations of orthogonalized signals (Hipp et al., 2012). We performed pairwise orthogonalization at each point in time before correlation. As a measure of phase coupling, we applied the weighted phase lag index (Vinck et al., 2011), which only takes into account the imaginary part of the cross-spectrum normalized by the average absolute imaginary contribution within the time series.

To characterize the temporal relationship between neuronal coupling and pupil size, we used a jackknife procedure to estimate the time course of neuronal coupling. For each pair of sources, we computed both coupling measures for all data and for all data but one window of 0.5 s. Then we computed coupling in the left-out window *C_i_* according to the following:

$$C_{i}=NC-\left(N-1\right)C_{-i},$$

where *N* is the number of nonoverlapping 0.5 s windows, *C* is the coupling computed for all data, and *C_–i_* is the coupling for the data without the 0.5 s window *i*. Amplitude coupling values were Fisher *z* transformed. For each source, we averaged the time courses of coupling to all other sources.

### Cross-correlation.

For all 457 sources, 35 frequencies and three neuronal measures (power envelope, phase coupling, amplitude coupling), we computed the temporal cross-correlation between pupil size and neuronal measures for temporal lags ranging from −20 to 20 s in 0.5 s steps. As the pupil was set as the temporal reference, negative latencies indicate a temporal precedence of power before pupil. The resulting correlation coefficients were Fisher *z* transformed for further statistics.

### Cross-ANOVA.

To capture nonlinear pupil-neuronal relationships we used a temporal cross-ANOVA. As for the cross-correlation approach, time series of power, phase, and amplitude coupling were shifted relative to each other from −20 to 20 s in 0.5 s steps. Pupil traces were then split into 10 bins of equal sample size. We then performed a one-way ANOVA with the factor pupil size for each neuronal measure of interest. For each source, frequency band, and lag, we calculated the corrected explained variance ω^2^ as the measure of effect size. For each source and frequency band, we also computed and subtracted the expected value of ω^2^ under the null hypothesis by circular shifting the neuronal time series with a random lag.

### Cluster permutation statistic.

To statistically assess cross-correlations or cross-ANOVAs between pupil size and power envelopes, phase coupling, or amplitude coupling, we conducted three-dimensional cluster-based permutation statistics (81 time lags × 35 frequencies × 457 sources; Hipp et al., 2011). First, for each time, frequency, and source we computed *t* values of correlation coefficients or unbiased ω^2^ across participants (*n* = 41). Next, we set all matrix entries with absolute *t* values exceeding the critical α of 1% to 1 and all other values to 0. We smoothed the resulting matrix by setting entries to 0 with <70% or 60% of neighbors with a 1 for power and coupling measures, respectively. We then defined continuous clusters of ones in the three-dimensional matrix and assigned to each cluster the integral of entailed *t* values as its size. We repeated the same procedure 1000 times, each time randomly flipping the sign of the correlation or the ω^2^ per subject. The *p* value of each original cluster was then assessed as the probability to find a cluster of that size or larger in the distribution of maximum cluster sizes across the 1000 permutations.

### Cross-correlation of cluster time courses with pupil dynamics.

For each identified cluster, we extracted the time course of log-power or coupling by weighted averaging of the original source-level time courses according to the ratio with which each frequency and source was included in the cluster at hand. We then cross-correlated these time courses with the time course of pupil size (see Figs. 3*C*, 4*C*, 5*C*, 6*C*), with the derivative of pupil size (see Figs. 3*C*, 4*C*, 5*C*, 6*C*), and with vectors indicating the time points of pupil maxima and minima (see Figs. 3*D*, 4*D*, 5*D*, 6*D*). For these cross-correlations, neuronal and pupil time courses were resampled with 10 ms resolution.

### Binning of neuronal and pupil data.

To characterize the relationship between neuronal measures and pupil size (see Figs. 3*E*, 4*E*, 5*E*, 6*E*), we computed neuronal measures as a function of the binned pupil size. For each cluster, we shifted the pupil size time courses relative to the neuronal time course according to the lag of maximum cross-correlation. We then averaged neuronal data and pupil sizes within 10 deciles of the pupil size data.

### Data availability.

Data and codes are available from the authors on request.

## Results

We recorded MEG data in human subjects (*n* = 41) during visual fixation at rest and simultaneously tracked gaze direction and pupil size (Fig. 1). To characterize the link between pupil size and band-limited neuronal activity in specific frequency ranges, we quantified the time course of band-limited signal power in 35 logarithmically spaced frequency bands from 0.6 to 215 Hz (0.5 octave bandwidth). To directly identify cortical correlates of pupil modulations, we reconstructed the time course of band-limited cortical population activity from the MEG data using beamforming (Fig. 1*B*; Van Veen et al., 1997; Gross et al., 2001). Time courses of both pupil size and band-limited population activity in different frequency bands showed an approximately power-law spectral profile, as indicated by the approximately linear relationship between the log-power and log-frequency (Fig. 1*B*).

In a first step, we correlated the time course of local cortical activity in different frequency bands (power envelope) with pupil dynamics. Neuronal activity could be related to pupil dynamics across substantial temporal lags (Reimer et al., 2016). Thus, we not only investigated the instantaneous correlation between neuronal activity and pupil dynamics, but also computed their cross-correlation across up to 20 s lags (Fig. 1*C*). Importantly, eye movements or related changes in luminance can induce changes in both pupil size and cortical activity. Indeed, we found that gaze eccentricity was significantly correlated with pupil size (*p* = 0.02, *t* test) and that gaze variance was correlated with occipitoparietal power changes across a broad frequency range. Thus, to exclude potentially confounding effects of eye movements, we partialled out gaze position and variance in all analyses relating pupil size to neuronal activity.

### Cortical population activity is tightly linked to pupil size

**Figure 2.**
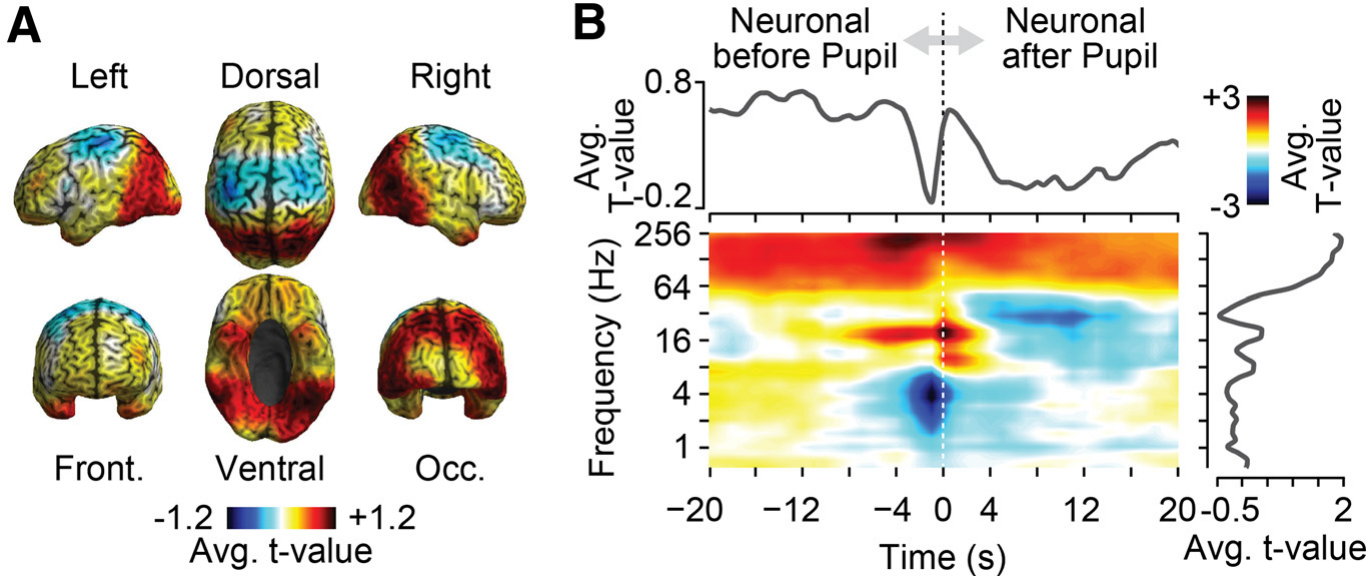
Cross-correlation of pupil size and local cortical population activity. The *t* values were computed across subjects (*n* = 41) as a function of cortical space, time, and frequency. ***A***, The *t* values averaged across time and frequency. ***B***, The *t* values averaged across cortical space (middle), across sources and frequencies (top), and across sources and time (right).

The cross-correlation analysis resulted in a large three-dimensional space (cortical space × time × frequency) of correlation between cortical activity and pupil size (Fig. 1*C*). As a first step, we performed a *t* statistic across subjects (random-effects) and marginalized this entire three-dimensional space onto different dimensions (Fig. 2). Specifically, we plotted the marginal *t* scores of correlation coefficients averaged across frequency and time on the brain (Fig. 2*A*), as well as averaged them over space as a function of time and frequency (Fig. 2*B*). These marginalized plots of the entire correlation space revealed a rich cortical pattern of positive and negative correlations at distinct temporal lags and frequencies.

Next, we used an unbiased approach (multidimensional cluster permutation statistic) to identify statistically significant spatially, spectrally, and temporally specific correlations of cortical activity with pupil dynamics (Fig. 1*C*; Maris and Oostenveld, 2007; Hipp et al., 2011). We identified four distinct cortical networks of local population activity that were significantly correlated with pupil dynamics (*p* < 0.05, corrected; permutation test).

### Frontal 4 Hz activity predicts pupil dilations

**Figure 3.**
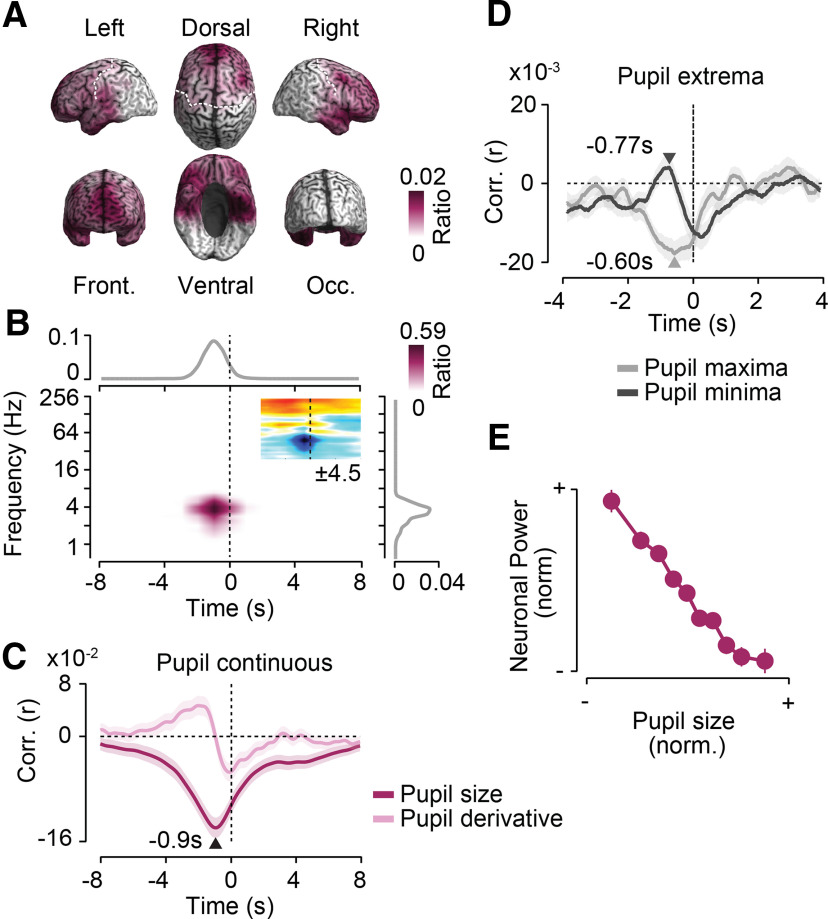
Frontal 4 Hz activity predicts pupil dilations. Results for a first cluster of negative cross-correlations between power and pupil size as shown. ***A***, Cortical distribution of in-cluster ratios across time and frequencies. For each cortical location, the in-cluster ratio is the ratio of time points and frequencies that are included in the cluster. ***B***, In-cluster ratios across sources (middle), across cortical locations and frequencies (top), and across locations and time (right). In-cluster ratios are the ratio of cortical locations (middle), locations and frequencies (top), and locations and time (right) that are included in the cluster. The small inset in the middle shows the time–frequency distribution of the cross-correlation of pupil size and cortical population activity within the frontal cluster. Colors indicate average *t* values across subjects (*n* = 41; maximum/minimum color scale of *t* values below inset; same time–frequency space as the main panel). ***C***, Temporal cross-correlation of in-cluster power with pupil size and with the temporal derivative of pupil size. ***D***, In-cluster power relative to time points of maximum and minimum pupil size expressed as a correlation coefficient. Arrows indicate the times of statistically compared peak correlations. ***E***, Average normalized pupil size and normalized in-cluster power within each 10% of the normalized pupil size. In-cluster power is shifted 0.9 s forward in time relative to pupil size. All shaded regions and error bars denote the SEM across subjects (*n* = 41).

A first anterior network (*p* = 0.009, corrected, permutation test) peaked in medial prefrontal cortex and extended into ventral frontal and temporopolar regions (Fig. 3*A*). Low-frequency activity at ∼4 Hz briefly before the pupil signal (range, −4.5 to 2 s) was anticorrelated with the following pupil signal (Fig. 3*B*). Thus, decreased low-frequency activity within this frontal network temporally predicted pupil dilations.

To investigate this neuronal–pupil relation in more detail, we extracted the time course of neuronal activity within the identified frequency range and frontal network. We cross-correlated this neuronal time course with pupil size (Fig. 3*C*, dark color) with the derivative of pupil size (Fig. 3*C*, bright color) and with the time points of peak pupil constriction or dilation (Fig. 3*D*; see Materials and Methods; Reimer et al., 2016).

This analysis revealed a maximum negative correlation between frontal low-frequency activity and pupil size for neuronal activity preceding pupil size by 0.9 s (Fig. 3*C*, dark color). The temporal profile of the correlation of frontal activity with the temporal derivative of pupil size (Fig. 3*C*, bright color) is the negative derivative of correlation with the raw pupil size. Thus, the temporal profile as such is not informative. However, the relative strength between the correlation with the raw pupil and with its derivative is informative as it depends on the spectral profile of the pupil signal (e.g., slow drifts may mask fast correlations with the raw pupil signal). We found that the peak correlation of neuronal activity with the pupil derivative was significantly smaller than with the raw pupil size (*p* < 10^−5^, *t* test). Thus, low-frequency power showed a tighter coupling to the raw pupil size than to its derivative.

The anticorrelation between neuronal power and pupil size could reflect increases of power preceding pupil constrictions and decreases of power preceding pupil dilations. Primarily, we found evidence for the latter scenario. Low-frequency power predicted pupil extrema shortly before both peak constrictions and dilations (Fig. 3*D*). However, the decrease of power 0.6 s before maximum pupil dilations was significantly stronger than the power increase 0.77 s before maximum pupil constrictions (*p* = 0.017, *t* test).

To further characterize the nature of anticorrelation, we shifted the pupil size by 0.9 s and averaged the normalized frontal low-frequency power within each decile of normalized pupil size (Fig. 3*E*). This revealed a monotonic relationship between pupil size and frontal low-frequency power that was almost linear across a wide dynamic range with smaller power changes for the most dilated pupil sizes. In summary, we identified a network of prefrontal regions in which decreases of ∼4 Hz population activity predicted pupil dilations of 0.9 s later.

### Precentral 16–32 Hz activity is suppressed before and after pupil dilations

**Figure 4.**
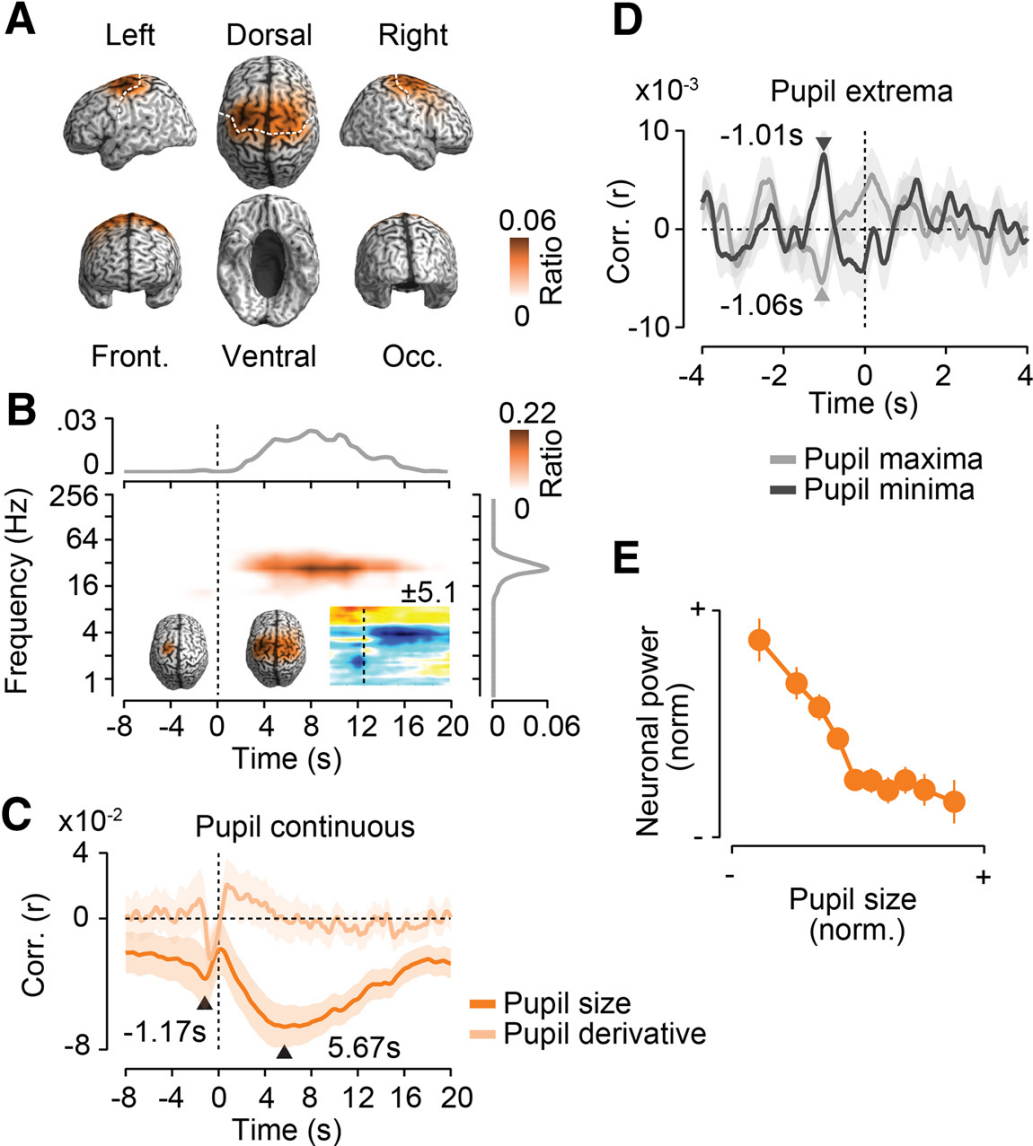
Precentral 16–32 Hz activity is suppressed before and after pupil dilations. Results for a second cluster of negative cross-correlations between power and pupil size. ***A***, Cortical distribution of in-cluster ratios averaged across time and frequencies. For each cortical location, the in-cluster ratio is the ratio of time points and frequencies that are included in the cluster. ***B***, In-cluster ratios averaged across cortical locations (middle), across locations and frequencies (top), and across locations and time (right). In-cluster ratios are the ratio of cortical locations (middle), locations and frequencies (top), and locations and time (right) that are included in the cluster. The small time–frequency inset shows the time–frequency distribution of the cross-correlation of pupil size and cortical population activity within the precentral cluster. Colors indicate average *t* values across subjects (*n* = 41; maximum/minimum color scale of *t* values above the inset; same time–frequency space as the main panel). The inset brains show the cortical distribution of in-cluster ratios for cross-correlations earlier (left inset) and later (right inset) than 1.5 s. ***C***, Temporal cross-correlation of in-cluster power with pupil size and with the temporal derivative of pupil size. ***D***, In-cluster power relative to time points of maximum and minimum pupil size expressed as a correlation coefficient. Arrows indicate the times of statistically compared peak correlations. ***E***, Average normalized pupil size and normalized in-cluster power within each 10% of the normalized pupil size. In-cluster power is shifted 5.67 s backward in time relative to pupil size. All shaded regions and error bars denote the SEM across subjects (*n* = 41).

We identified a second cluster of significant anticorrelation (*p* = 0.005, corrected, permutation test) between cortical population activity and pupil size (Fig. 4). This cluster involved bilateral precentral regions compatible with premotor cortex, supplementary motor areas, and the frontal eye fields (FEFs; Fig. 4*A*). In this cortical network, population activity at ∼16–32 Hz prominently decreased with larger pupil size for ∼18 s directly following the pupil signal (lag of peak anticorrelation, 5.67 s; Fig. 4*B*,*C*). Furthermore, there was a brief decrease of 16–32 Hz power 1.17 s before larger pupil sizes (Fig. 4*B*,*C*). The cortical distribution of the pupil preceding effect was spatially more confined, but was generally similar to the cortical distribution of the pupil following effect (Fig. 4*B*, insets). As for the pupil predictive 4 Hz frontal network, the pupil showed a significantly stronger correlation with pupil size than with the derivative of pupil size (*p* = 0.016, *t* test).

Triggering power in the identified precentral network and frequency range on pupil extrema (Fig. 4*D*) revealed pupil-predictive power ∼1 s before peak pupil constrictions and dilations. There was no significant difference between the strength of the effect preceding constrictions and dilations (*p* = 0.66, *t* test). Notably, the long-lasting power decrease following pupil dilations was not depicted in the extrema-triggered analysis, suggesting that these longer lasting pupil dilations averaged out in the peak-triggered analysis. The relationship between precentral power following binned pupil size by 5.67 s showed a slightly nonlinear monotonic anticorrelation with stronger power changes in the constricted pupil regimen (Fig. 4*E*).

In summary, 16–32 Hz activity in a well confined bilateral precentral network briefly decreased 1.17 s before pupil dilations and showed a long-lasting suppression for up to 20 s following pupil dilations.

### Posterior 16 Hz activity increases before pupil dilations

**Figure 5.**
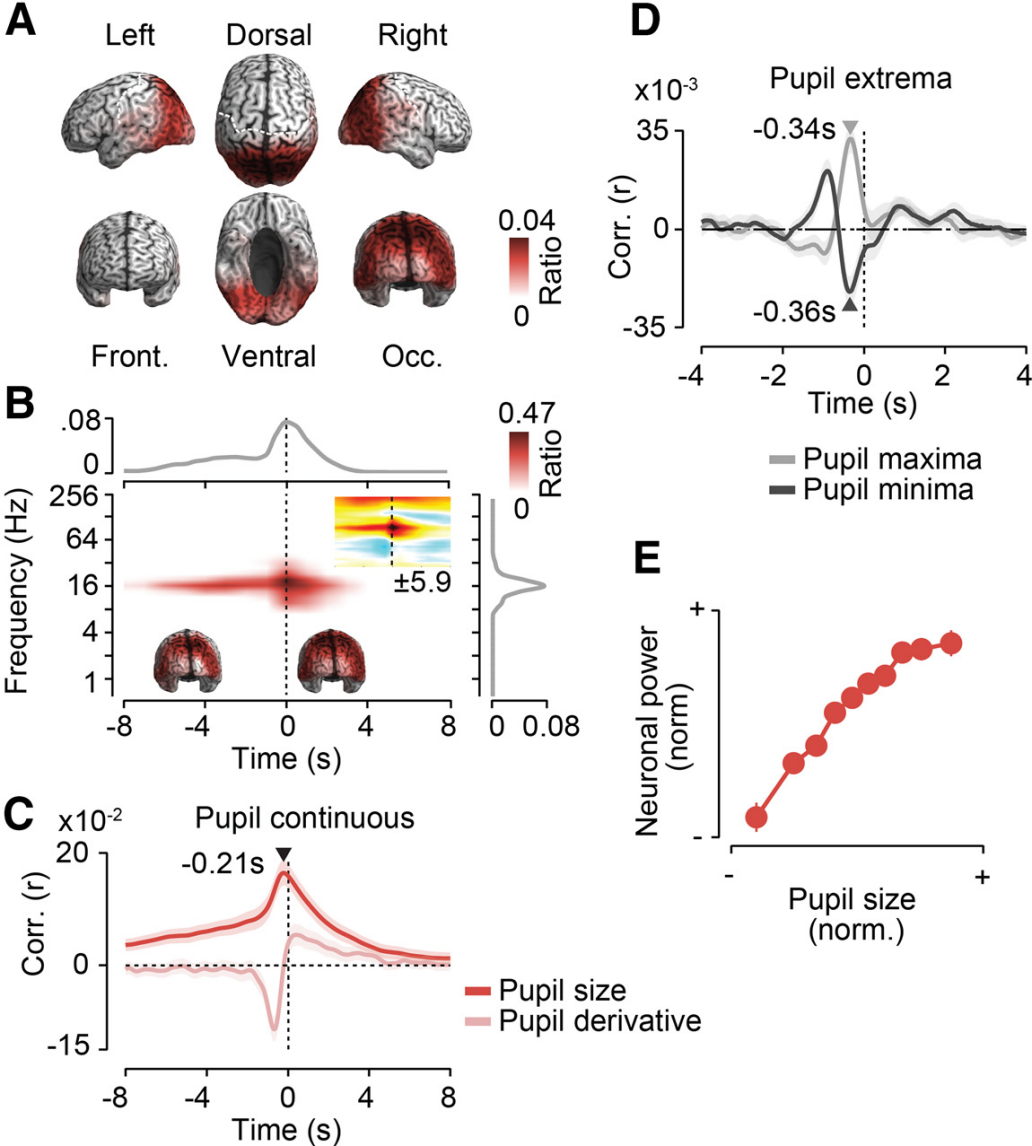
Posterior 16 Hz activity increases before pupil dilations. Results for a first cluster of positive cross-correlations between power and pupil size are shown. For each cortical location, the in-cluster ratio is the ratio of time points and frequencies that are included in the cluster. ***A***, Cortical distribution of in-cluster ratios averaged across time and frequencies. ***B***, In-cluster ratios averaged across cortical locations (middle), across locations and frequencies (top), and across locations and time (right). In-cluster ratios are the ratios of cortical locations (middle), locations and frequencies (top), and locations and time (right) that are included in the cluster. The small time–frequency inset shows the time–frequency distribution of the cross-correlation of pupil size and cortical population activity within the precentral cluster. Colors indicate average *t* values across subjects (*n* = 41; maximum/minimum color scale of *t* values below the inset; same time–frequency space as the main panel). The inset brains show the cortical distribution of in-cluster ratios for cross-correlations earlier (left inset) and later (right inset) than −1.5 s. ***C***, Temporal cross-correlation of in-cluster power with pupil size and with the temporal derivative of pupil size. ***D***, In-cluster power relative to time points of maximum and minimum pupil size expressed as a correlation coefficient. Arrows indicate the times of statistically compared peak correlations. ***E***, Average normalized pupil size and normalized in-cluster power within each 10% of the normalized pupil size. In-cluster power is shifted 0.21 s forward in time relative to pupil size. All shaded regions and error bars denote the SEM across subjects (*n* = 41).

Third, we found a positive correlation between pupil size and power at ∼16 Hz in parietal and occipitotemporal regions (*p* = 0.007, corrected, permutation test; Fig. 5*A*,*B*). Power increases preceded pupil dilations by up to 8 s, peaked 0.21 s before the pupil, and followed pupil changes for up to 4 s (Fig. 5*B*,*C*). Early and late components of pupil predictive activity had similar cortical profiles (Fig. 5*B*, insets).

Again, power better predicted absolute pupil size than its temporal derivative (*p* < 10^−5^, *t* test; Fig. 5*C*). Posterior power predicted both peak power dilations and constrictions (Fig. 5*D*), with no significant difference between both effects (*p* = 0.21, *t* test). Averaging the posterior 16 Hz power as a function of pupil size (0.21 s lag) revealed a nonlinear monotonic relationship with the strongest power changes for smaller pupil sizes (Fig. 5*E*). In summary, 16 Hz activity in parietal and occipitotemporal regions was enhanced before and around pupil dilations.

The cluster statistics of the cross-correlation between pupil size and power revealed a fourth significant cluster (*p* = 0.001, corrected, permutation test) for high-frequency power >64 Hz (Figs. 2B, 3*B*, 4*B*, 5*B*). Power in this frequency range was positively correlated with pupil size in a widespread network of occipital, parietal, and ventral regions with latencies ranging from −20 to 20 s. MEG power in this high-frequency range is prone to reflect muscle activity, and the reconstructed cortical profile was compatible with projected muscle artifacts. Thus, we refrained from interpreting this finding as a cortical neuronal correlate of pupil dynamics. In addition, we report a fifth cluster that did not reach statistical significance (*p* = 0.12). For this cluster, dorsal frontal activity at ∼64 Hz increased ∼1.27 s before pupil dilations.

### Large-scale cortical amplitude coupling predicts pupil dynamics

The above results unravel a rich set of local modulations of band-limited activity that predicted or followed pupil dynamics. In a next step, we investigated whether pupil dynamics were also associated with modulations of the large-scale coupling of neuronal activity. Again, we quantified neuronal coupling in a frequency-specific manner. We investigated the following two distinct modes of coupling (Siegel et al., 2012; Siems and Siegel, 2020): phase coupling, which quantifies the phase consistency between band-limited activity in different regions; and amplitude coupling, which quantifies the correlation of the envelopes of band-limited activity in different regions (Fig. 1*B*). Importantly, for both coupling modes, we used measures that discount any spurious correlations because of field-spread effects (i.e., amplitude correlation of orthogonalized signals and a weighted phase-lag index; Vinck et al., 2011; Hipp et al., 2012). We used the same cluster permutation statistic as that for power described above to pinpoint brain regions, the coupling of which was correlated with pupil size at a specific temporal lag and frequency of neuronal activity (Fig. 1*C*; for details, see Materials and Methods).

**Figure 6.**
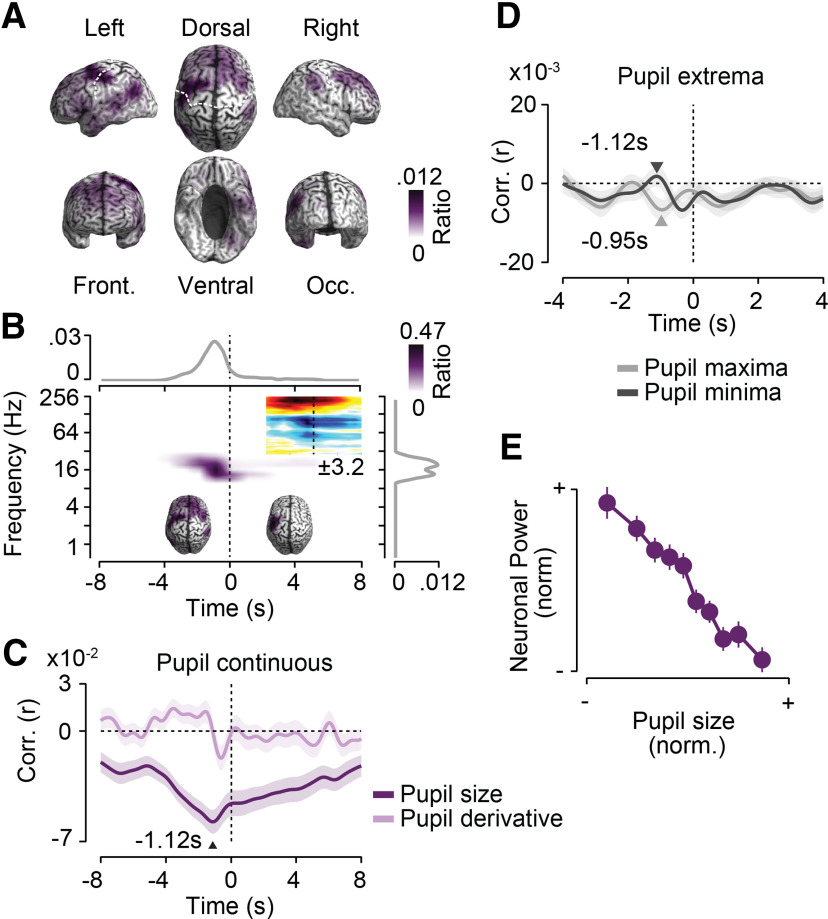
Large-scale cortical amplitude coupling predicts pupil dynamics. Results for a first cluster of negative cross-correlations between corticocortical amplitude coupling and pupil size are shown. ***A***, Cortical distribution of in-cluster ratios averaged across time and frequencies. For each cortical location, the in-cluster ratio is the ratio of time points and frequencies that are included in the cluster. ***B***, In-cluster ratios averaged across cortical locations (middle), across locations and frequencies (top), and across locations and time (right). In-cluster ratios are the ratio of cortical locations (middle), locations and frequencies (top), and locations and time (right) that are included in the cluster. The small time–frequency inset shows the time–frequency distribution of cross-correlation of pupil size and cortical population activity within the precentral cluster. Colors indicate average *t* values across subjects (*n* = 41; maximum/minimum color scale of *t* values below inset; same time–frequency space as the main panel). The inset brains show the cortical distribution of in-cluster ratios for cross-correlations earlier (left inset) and later (right inset) than 2 s. ***C***, Temporal cross-correlation of in-cluster amplitude coupling with pupil size and with the temporal derivative of pupil size. ***D***, In-cluster amplitude coupling relative to time points of maximum and minimum pupil size expressed as a correlation coefficient. Arrows indicate the times of statistically compared peak correlations. ***E***, Average normalized pupil size and normalized in-cluster amplitude coupling within each 10% of the normalized pupil size. In-cluster amplitude coupling is shifted 1.12 s forward in time relative to pupil size. All shaded regions and error bars denote the SEM across subjects (*n* = 41).

For amplitude coupling, we identified a large-scale network of frontal and left parietal brain regions in which amplitude coupling from ∼10 to 32 Hz was significantly anticorrelated with pupil size (*p* = 0.022, corrected, permutation test; Fig. 6). The anticorrelation peaked at −1.12 s and extended into positive lags (Fig. 6*B*,*C*). Thus, changes in network coupling preceded pupil dynamics and followed these. The cortical distribution of the pupil-predictive coupling changes was widespread, including frontal and parietal regions (Fig. 6*B*, left brain, inset). In contrast, the later coupling modulation at ∼27 Hz was more focused on left precentral regions, which well resembled the cortical location of the precentral power changes predicting pupil dynamics (compare Figs. 6*B*, right brain inset, 4*B*, left brain inset). As for the power changes, amplitude coupling showed a stronger correlation with pupil size than with the derivative of pupil size (*p* < 10^−4^, *t* test; Fig. 6*C*). Triggering the time course of amplitude coupling on peak pupil constrictions and dilations did not reveal well dissociable coupling peaks (Fig. 6*D*). Average amplitude coupling as a function of binned pupil size (−1.12 s lag) showed a monotonic approximately linear relationship (Fig. 6*F*).

We identified a second significant cluster of positive correlation of amplitude coupling and pupil size in the high gamma-band >64 Hz (Fig. 6*B*, middle, time–frequency inset). Similar to the power effects in the high-gamma band, the spectral extent and cortical distribution of this coupling effect was reminiscent of residual muscle artifacts. Thus, we refrained from interpreting this effect in terms of neuronal coupling.

We applied the same analysis approach used for amplitude coupling to phase coupling. In contrast to amplitude coupling, there was no significant correlation between cortical phase coupling and pupil size (*p* > 0.05).

### Absence of nonmonotonic effects

Neuromodulation may have nonmonotonic, e.g. U-shaped effects on neuronal population activity and interactions (Aston-Jones and Cohen, 2005; McGinley et al., 2015a,b). Such modulations could have been missed by the above-described analysis approach, which is based on linear correlation. Thus, we repeated the entire analysis, again using cluster permutation statistics but based on a cross-ANOVA approach that is sensitive to any type of dependency between neuronal and pupil data across temporal lags (see Materials and Methods).

For the power of local population activity, this resulted in one significant cluster (*p* < 0.001, corrected, permutation statistic). This cluster matched the above occipitoparietal cluster with a positive sublinear but monotonic prediction of 16 Hz power by pupil size (Fig. 5). There was no other significant cluster (*p* > 0.01).

We repeated the same cross-ANOVA-based analysis for phase and amplitude coupling. This did not yield any significant effect. Thus, we concluded that there were no significant nonmonotonic relationships between pupil dynamics and local neuronal population activity, phase coupling, or amplitude coupling.

### Control analyses

We performed several control analyses to assess the robustness of the above findings. Our sample included one group of subjects that fixated a colored dot and a second group that fixated the middle of a blank screen. To ensure that our effects were not specific to only one of the groups, we separately tested all main effects for both groups (Figs. 3–6, neuronal–pupil correlation at peak lag). All main effects were significant in both groups (all *p* < 0.01). Thus, the reported effects were not dependent on the specific fixation target.

In all of the above analyses, we partialled out gaze eccentricity and gaze variance. To ensure that our results were not confounded by this partialization, we tested all main effects (Fig. 3–6, correlation at peak lag) without partializing out gaze eccentricity and gaze variance. All effects were significant without the partialization (all *p* < 0.001) and were thus not confounded by accounting for gaze eccentricity and gaze variance.

For all above analyses, no high-pass filter was applied to the pupil signal, which thus contained slow drifts across several minutes as well as fast dynamics on the time scale of seconds. To assess to what extent slow drifts affected our results and potentially masked neuronal relations to faster pupil dynamics, we repeated the cross-correlation and cluster permutation statistic for local population activity after high-pass filtering pupil time courses using a 0.01 Hz cutoff frequency. This analysis yielded the same cortical networks as the original analysis, for which we had not applied a high-pass filter. Moreover, we did not identify any additional significant networks in this new analysis. Thus, the identified effects were primarily driven by dynamics faster than 0.01 Hz, and slower fluctuations did not mask additional effects.

## Discussion

**Figure 7.**
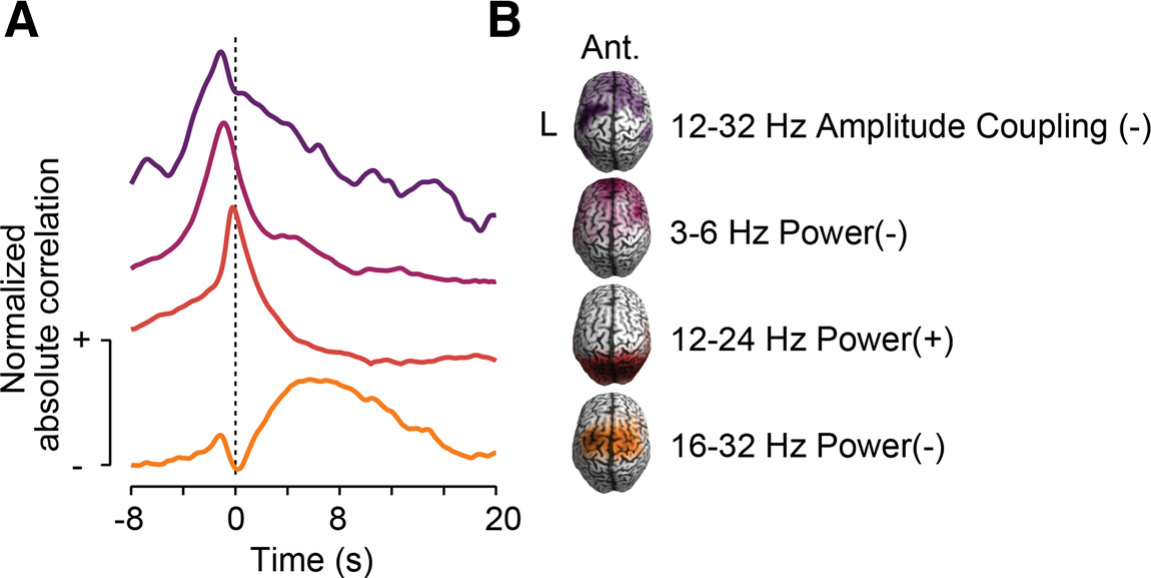
Summary of results. ***A***, Time course of normalized absolute cross-correlation of band-limited power or amplitude coupling of the four identified cortical networks that predict pupil size. ***B***, Cortical distribution of in-cluster ratios, approximate frequency range, type of neuronal population activity and sign of cross-correlation between neuronal activity and pupil size for the four identified cortical networks that predict pupil size.

Our results provide a systematic, time-resolved, and brainwide characterization of band-limited cortical correlates of pupil-linked neuromodulation in the human brain. We identified four highly specific cortical networks of band-limited local activity and large-scale coupling that temporally predicted and followed non-luminance-mediated pupil dynamics (Fig. 7).

Our results are based on an unbiased analytical approach. Specifically, the use of a cluster-based permutation statistic did not require any a priori binning into frequency bands or the preselection of cortical regions of interest (Maris and Oostenveld, 2007; Hipp et al., 2011). Furthermore, we directly conducted all analyses in cortical source space, partialled out the potential confounding effects of eye movements, and used phase- and amplitude-coupling measures that discount spurious coupling because of field-spread effects (Vinck et al., 2011; Hipp et al., 2012). This allowed for a comprehensive and unbiased characterization of the cortical correlates of pupil dynamics across the entire human cortex, a broad frequency range (0.6–215 Hz), and lags of up to 20 s.

### Spectral and spatial specificity

Our results bridge a vast gap between animal and human experiments investigating neuromodulation. Recent findings in mice and nonhuman primates show modulations of the spectral profile of LFP activity with pupil dynamics (Reimer et al., 2014, 2016; McGinley et al., 2015a,b; Vinck et al., 2015; Joshi et al., 2016). In these studies, pupil dilations have been associated with increases of fast population dynamics (>30 Hz) and decreases of slow population dynamics (<10 Hz), particularly in sensory cortical regions. Also recent human MEG and electroencephalography studies that used an instantaneous correlation of neuronal activity and pupil size found a suppression of slow activity (<4–8 Hz) and enhanced fast activity (∼14–90 Hz) around the time of pupil dilation in auditory and further cortical regions (Waschke et al., 2019; Podvalny et al., 2021; Pfeffer et al., 2022). Our results are in principle compatible with these findings. We also found widespread increases of high gamma-band activity with pupil dilation, which may, however, be confounded by muscle artifacts, as well as decreases of slow activity around pupil dilations in several regions (compare Figs. 2*B*, 3*B*, 4*B*, 5*B*, time–frequency insets). However, our brainwide characterization of the cortical correlates of pupil dynamics across a broad temporal scale also uncovered more specific spectral and spatial patterns of cortical neuromodulation.

Thus, our results show that pupil dilations are not merely associated with a generic antagonistic modulation of low- and high-frequency activity throughout the cortex, but that there are modulations in several confined cortical networks with spectral and temporal profiles that are specific for the cortical network at hand. This is also highlighted by the distinct spectrotemporal profiles of unmasked pupil-linked cortical population activity associated with the different cortical networks (Figs. 3*B*, 4*B*, 5*B*, 6*B*, small time–frequency insets). Furthermore, our findings accord well with those from another recent MEG study (Pfeffer et al., 2022) that, based on a different analytical approach, also found correlations between pupil size and neural activity compatible with the frontal 4 Hz and posterior 16 Hz networks described here.

The spectral marginals of the identified cortical networks as well as the unmasked time–frequency distribution of effects show that all identified effects have a bandwidth (full-width at half-maximum) of ∼1 octave. The demonstrated effects are thus spectrally specific. Furthermore, our results show that, with the exception of the frontal 4 Hz effect, pupil-related modulations potentially are sustained across several seconds, which is multiple times the period of the corresponding frequencies. Thus, pupil-related power changes extend across many cycles of spectrally specific signals. In principle, our results are neutral concerning the question of whether, beyond power, the phase of the pupil-predictive neuronal activity also is time locked to pupil size. While components of the demonstrated pupil-linked signals may thus be phase locked to pupil dynamics, the spectrotemporal properties of the identified signals are incompatible with only transient broad-band signals that are phase locked to the pupil dynamics explaining these effects. Furthermore, the temporally more confined 4 Hz effect in frontal regions also does not spectrally match the MEG signals that have been described as phase locked to fast pupil dynamics (Podvalny et al., 2021).

The uncovered cortical networks and frequencies that were linked to pupil dynamics resemble characteristic cortical networks of rhythmic population activity. Our finding that widespread prefrontal theta-band activity predicts pupil dynamics (Fig. 3) accords well previous findings that implicate prefrontal theta-band activity in cognitive control processes (Jensen and Tesche, 2002; Rutishauser et al., 2010; Cavanagh and Frank, 2014). Also, the observed pupil-linked modulations of beta-band activity in precentral regions (Fig. 4) well resemble previous findings of beta-band modulations in the prefrontal cortex and specifically FEF associated with visual attention (Buschman and Miller, 2007; Siegel et al., 2008, 2012; Hipp et al., 2011; Gregoriou et al., 2012; Babapoor-Farrokhran et al., 2017). Finally, the pupil-linked modulations of ∼16 Hz in occipitoparietal cortex (Fig. 5) match the well known predominance and cognitive modulation of rhythmic activity in this frequency range in occipitoparietal regions (Siegel et al., 2008; Van Der Werf et al., 2008; Donner and Siegel, 2011; Hipp et al., 2012). Importantly, these different pupil-linked effects show distinct temporal profiles and different signs of relation to pupil size. Thus, the reported effects cannot be explained as merely reflecting an unspecific pupil-linked effect in combination with the variable strength of band-limited activity in different cortical networks. Instead, these results suggest that pupil-linked neuromodulation is specifically related to several well known cortical networks of rhythmic brain activity that have typically been implicated in attention and cognitive control. In turn, our results suggest that previously observed modulations of band-limited activity within these networks may at least partially reflect neuromodulatory effects that also drive pupil fluctuations. This accords well with recent studies showing that pupil-linked modulations of band-limited neuronal population activity predict human sensory processing and performance (Waschke et al., 2019; Podvalny et al., 2021).

Previous studies indicated an inverted-U shaped relation between pupil size and perceptual behavior (McGinley et al., 2015b; Waschke et al., 2019; Podvalny et al., 2021) as well as between pupil size and population activity at ∼10 Hz (Podvalny et al., 2021; Pfeffer et al., 2022). Our time-resolved analysis did not yield such a strictly nonmonotonic relationship between cortical activity and pupil dynamics. Nevertheless, we found that posterior 16 Hz activity sublinearly increased with pupil size, which well resembled the lower half of the inverted-U shape. Thus, differences in the sampled range of arousal and pupil dynamics may explain these results.

### Large-scale neuronal coupling

In addition to pupil-linked modulations of local population activity, for the first time, to our knowledge, our results uncover pupil-linked modulations of large-scale corticocortical coupling. Notably, large-scale phase coupling, which is modulated by various cognitive processes (Engel et al., 2001; Siegel et al., 2008, 2012; Fell and Axmacher, 2011) and has been implicated in neuronal communication (Fries, 2015), was not linked to spontaneous pupil dynamics. In contrast, frontoparietal amplitude coupling at ∼16 Hz predicted pupil dilations (Fig. 6). Cortical amplitude coupling is frequency specific, spatially well structured and dissociated from phase coupling (Hipp et al., 2012; Siems and Siegel, 2020). Amplitude coupling resembles fMRI correlation patterns (Mantini et al., 2007; Hipp and Siegel, 2015), and is largely driven by slow fluctuations <0.1 Hz (Hipp et al., 2012). These slow fluctuations are well compatible with neuromodulatory effects. In accordance with this hypothesis, our results show that cortical amplitude coupling is linked to cortical neuromodulation as indexed by pupil dynamics. Furthermore, our results provide further evidence that phase and amplitude coupling are independent coupling modes that reflect at least partly distinct underlying neuronal mechanisms (Siegel et al., 2012; Engel et al., 2013; Siems and Siegel, 2020).

### Cause and effects

Temporally resolving the relationship between cortical activity and pupil dynamics provided important insights into their potential causal relationship. All four local and large-scale cortical networks that we identified showed cortical modulations that temporally preceded pupil dynamics (Fig. 7). For all networks, the lag of peak predictive correlation was between 0.2 and 1.2 s. This accords well with the delay between LC stimulation and pupil dilations in primates (∼0.5 s; Joshi et al., 2016) as well as with the delay between noradrenergic (∼1 s) and cholinergic (∼0.5 s) axon activations and pupil dilations in mice (Reimer et al., 2016). This suggests that pupil-linked activity within the identified cortical networks is temporally tightly linked to the subcortical neuromodulatory centers. The temporal precedence together with the functional role of the identified frontoparietal regions in cognitive control processes suggests that these cortical networks may in fact play a causal role for controlling neuromodulatory signals. Manipulative approaches will be required to decisively test this hypothesis.

All identified networks also showed pupil-linked neuronal activity that coincided with or lagged the pupil size. The brief positive lags of neuronal activity in frontal (Figs. 3, 6) and occipitoparietal (Fig. 5) networks are well compatible with the slow time constant of noradrenergic and cholinergic axonal activations that also show positive correlations following the pupil signal, in particular for the cholinergic system (Reimer et al., 2016). In contrast to these brief effects, precentral 16–32 Hz activity showed much slower and more lagged dynamics (Fig. 4). In this network, in addition to a brief pupil-predictive peak, the relation between neuronal activity and pupil size peaked 5 s following the pupil signal and lasted for up to 18 s. This slow and delayed link to the pupil signal suggests that this late precentral activity may reflect the effect of neuromodulation, rather than its cause.

In addition to these physiological implications, our results highlight that a mere instantaneous correlation analysis is unable to unravel the complex and temporally lagged relationship between neuronal activity and pupil dynamics.

### Future directions

Our findings set the stage for several lines of future research. First, we took a conservative approach not interpreting pupil-linked modulations of power and amplitude coupling in the high gamma band because of likely confounding by muscle artifacts. Invasive microelectrode or electrocorticogram recordings may allow the unequivocal addressing of this question. Second, our results identify pupil-linked dynamics of neuronal activity on the population level. It will be important to link these findings to neuronal activity and interactions on the cellular and spiking levels using invasive recordings. Invasive recordings and pharmacological approaches may also allow the specific linking of the demonstrated effects to the noradrenergic or cholinergic systems. Third, our findings identify potential cortical neuromodulatory control networks. This provides a basis for manipulative approaches in future animal and human experiments to probe the causal contributions of these cortical networks. Fourth, pupil-associated neuromodulation has been implicated in various cognitive functions such as arousal, decision-making, and exploration-exploitation preference (Kahneman and Beatty, 1966; Einhäuser et al., 2008, 2010; Gilzenrat et al., 2010; Jepma and Nieuwenhuis, 2011; Preuschoff et al., 2011; Nassar et al., 2012; Alnæs et al., 2014; de Gee et al., 2014; Pajkossy et al., 2017; Urai et al., 2017; Waschke et al., 2019; Podvalny et al., 2021). It will be important to investigate how the cortical correlates of pupil-linked neuromodulation that were identified here during rest generalize to behavioral contexts specifically probing these cognitive processes. Along the same line, arousal fluctuations pose a prominent confounder in many experiments. Our results indicate that pupil fluctuations may allow the accounting of a substantial amount of variance in cortical network activity driven by this confounder. Finally, investigating disturbances of pupil fluctuations and their cortical correlates identified here could provide powerful biomarkers of neuromodulatory system abnormalities.

### Conclusions

Our results provide a systematic characterization of time-resolved, band-limited cortical correlates of pupil-linked neuromodulation in the human brain. We found that pupil-linked neuromodulation does not merely affect cortical population activity in a stereotypical fashion. Instead, we found that cortical activity and coupling is tightly linked to pupil dynamics in several temporally, spectrally, and spatially specific networks. Neuronal effects temporally preceded and followed pupil dynamics, which likely reflects the cortical causes and effects of neuromodulation. Our results open a powerful new window to noninvasively investigate the mechanisms of cortical neuromodulation in the healthy and diseased human brain during cognition and behavior.
